# Correlation between in-brace radiographic correction and short time brace results

**DOI:** 10.1186/1748-7161-7-S1-O27

**Published:** 2012-01-27

**Authors:** F Zaina, S Donzelli, M Lusini, S Negrini

**Affiliations:** 1 ISICO Milan, Italy; 2 Siena University, Italy

## Background and purpose

In-brace X-ray is considered a reliable check of brace efficacy [1-3]. The aim of this study was to correlate the in-brace correction with the short term results of treatment (6 months).

## Materials and methods

Design: pre-post study.

Population: 41 consecutive adolescent girls with idiopathic scoliosis who were prescribed a brace treatment (39 thoracic curves, 37±12°; 16 thoracolumbar, 38±13°; 12 lumbar, 31±8°. Risser 0-3).

In-brace X-ray and 6 months treatment out of brace X-ray results were correlated, according to curve localization. The in-brace/out-of-brace ratio was calcutated, curves were grouped according to the Risser sign, the results (<10°,³10° out-of-brace), in-brace correction (<10°,³10°), the magnitude (<30°, 30°-45°, >45°).

Statistical analysis: Correlation Coefficient.

## Results

The in-brace/out-of-brace ratio varied according to localization of curve and Risser, achieving the best results for Thoracic curves (38-45%). The groups of Thoracolumbar and Lumbar had higher variability (17-65% and 17-40%). The correlation coefficient between in-brace correction and out of brace results was statistically significant: 0.85 for Thoracic curves, 0.64 thoracolumbar, 0.72 lumbar. Risser groups: 0.65-0.98 Thoracic, 0,78-0.90 Thoracolumbar, 0.94-0.98 Lumbar. For Results groups, the correlation was better for High results in lumbar and Low results for thoracolumbar, no differences for thoracic. Low in-brace correction had a low correlation coefficient for thoracic and lumbar curves. No differences for Magnitude.

## Conclusions

The correction after 6 months of brace are 17-47% of the in-brace correction. The correlation between in-brace correction and short time results of brace is significant, range 0.64-0.98. The in-brace correction seems able to predict the short time results of treatment.
